# 4-cholesten-3-one decreases breast cancer cell viability and alters membrane raft-localized EGFR expression by reducing lipogenesis and enhancing LXR-dependent cholesterol transporters

**DOI:** 10.1186/s12944-019-1103-7

**Published:** 2019-09-02

**Authors:** Josiane Elia, Delphine Carbonnelle, Cédric Logé, Lucie Ory, Jean-Michel Huvelin, Mona Tannoury, Mona Diab-Assaf, Karina Petit, Hassan Nazih

**Affiliations:** 1 Faculté des Sciences Pharmaceutiques et Biologiques, Université de Nantes, 9 Rue Bias, BP 53508, F-44035 Nantes Cedex 1, France; 2 Département de Chimie Thérapeutique, Université de Nantes, Nantes Atlantique Universités, EA1155 - IICiMed, Faculté de Pharmacie, Nantes, France; 3 Faculté des Sciences II, Ecole Doctorale des Sciences et de Technologie, Université Libanaise, Fanar, Lebanon

**Keywords:** 4-cholesten-3-one, Breast cancer cells, Lipogenesis, LXR, Cholesterol efflux, Membrane raft

## Abstract

**Background:**

The alteration of lipid metabolism in cancer cells is recognized as one of the most important metabolic hallmarks of cancer. Membrane rafts defined as plasma membrane microdomains enriched in cholesterol and sphingolipids serve as platforms for signaling regulation in cancer. The main purpose of this study was to evaluate the effect of the cholesterol metabolite, 4-cholesten-3-one, on lipid metabolism and membrane raft integrity in two breast cancer cell lines, MCF-7 and MDA-MB-231. Its ability to reduce cell viability and migration has also been investigated.

**Methods:**

RT-qPCR was performed to evaluate the expression of enzymes involved in lipogenesis and cholesterol synthesis, and ABCG1 and ABCA1 transporters involved in cholesterol efflux. Its effect on cell viability and migration was studied using the MTT assay, the wound healing assay and the Transwell migration assay, respectively. The effect of 4-cholesten-3-one on membrane rafts integrity was investigated by studying the protein expression of flotillin-2, a membrane raft marker, and raft-enriched EGFR by western blot.

**Results:**

Interestingly, we found that 4-cholesten-3-one treatment decreased mRNA expression of different enzymes including ACC1, FASN, SCD1 and HMGCR. We further demonstrated that 4-cholesten-3-one increased the expression of ABCG1 and ABCA1. We also found that 4-cholesten-3-one decreased the viability of MCF-7 and MDA-MB-231 cells. This effect was neutralized after treatment with LXR inverse agonist or after LXRβ knockdown by siRNA. As a result, we also demonstrated that 4-cholesten-3-one disrupts membrane rafts and cell migration capacity.

**Conclusion:**

Our results show that 4-cholesten-3-one exerts promising antitumor activity by altering LXR-dependent lipid metabolism in breast cancer cells without increasing lipogenesis.

## Background

Breast cancer is the most commonly diagnosed cancer and the second leading cause of cancer death among women [1]. An estimated 2.1 million new cases of cancer and 627,000 deaths from breast cancer occurred in 2018 worldwide [2].

Cancer is typically characterized by abnormal and uncontrolled cell proliferation, resistance to apoptosis, cell migration, and other key features. On top of this, deregulation of lipid metabolism is known to be one of the emerging metabolic hallmarks of cancer cells [3]. It is known that rapidly proliferating cancer cells require a large amount of lipids for cell membrane synthesis and an increased need for energy [4]. To meet their lipid requirements, cancer cells exhibit increased lipogenesis (also known as synthesis of de novo fatty acids) [5]. Briefly, lipogenesis is initiated by carboxylation of acetyl-CoA to malonyl-CoA catalyzed by ACC (acetyl-CoA carboxylase) [6]. Then, FASN (fatty acid synthase) synthesizes palmitic acid from acetyl-CoA and malonyl-CoA. The desaturation of newly synthesized fatty acids is then catalyzed by SCD1 (stearoyl-CoA desaturase 1) which inserts a double bond in the Δ9 position of palmitic acid to produce monounsaturated fatty acids, palmitoleic acid. Another metabolic pathway of lipid metabolism is the mevalonate pathway, which synthesizes cholesterol [7]. HMGCR (3-hydroxy-3-methyl-glutaryl-coenzyme A reductase) is the rate-controlling enzyme of this pathway, it catalyzes the conversion of HMG-CoA to mevalonic acid, a necessary step in the biosynthesis of cholesterol [7]. It has been shown that the enzymes involved in lipogenesis and cholesterol biosynthesis are highly expressed in various cancer cells, such as breast and prostate cancer cells, and have recently been reported as a target for cancer treatment [6, 8].

The liver X receptor (LXR) is a transcription factor of the nuclear receptor superfamily that forms heterodimers with the retinoid X receptor (RXR) and can be activated with a natural or synthetic agonist. LXRα is expressed in all metabolically active tissues such as liver, adipose tissue, kidneys, intestine and macrophage, whereas LXRβ is expressed ubiquitously. Activated LXR regulates the expression of genes involved in lipogenesis such as ACC1, FASN, SCD1 and SREBP-1c (sterol regulatory element-binding protein-1c) and in cholesterol efflux, the first step of the reverse transport of cholesterol from macrophage to liver, such as ABCG1 (ATP-binding cassette sub-family G member 1), ABCA1 (ATP-binding cassette sub-family A member 1) and APOE (apolipoprotein E) [9, 10]. Recent studies have demonstrated the antiproliferative effect of LXR agonists in various types of cancers such as leukemia, prostate, breast, ovarian and colon cancers [11]. It has been reported that activation of LXR deprives MCF-7 breast cancer cell membranes of cholesterol essential for their growth by stimulating its efflux via ABCG1, resulting in inhibition of cell proliferation and induction of apoptosis [12].

Cholesterol is an important component of membrane rafts which are microdomains of the plasma membrane. These microdomains serve as membrane platforms for signaling molecules that are involved in a variety of cellular functions including cell growth, survival and migration, such as epidermal growth factor receptors (EGFRs) which are overexpressed in many cancer types such as breast and colon cancers [13, 14]. Therefore, it has been suggested that membrane rafts play a functional role during tumorigenesis [15]. Flotillin-2 is identified as a major protein on microdomain membrane rafts. Its expression has been shown to be upregulated in various cancer cells, including breast cancer cells, suggesting its involvement in carcinogenesis [16, 17]. In addition, it has been reported that its downregulation is associated with the inhibition of cancer cell proliferation, migration and invasion [18].

**Fig. 1:**
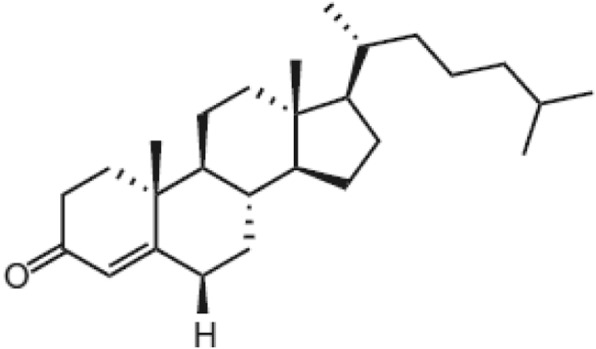
Chemical structure of 4-cholesten-3-one

In the gastrointestinal tract, cholesterol is metabolized to 4-cholesten-3-one (Fig. 1) by cholesterol oxidase [19]. This cholesterol derivative is identified in bile, blood, gallstones and human faeces [20, 21]. It can also be found in marine algae [22, 23], marine fish [24] and plant roots [25]. It should be noted that it was also identified by our team in an extract of a red marine alga *Laurencia papillosa* collected from the Lebanese coast (data not yet published). 4-cholesten-3-one exerts various biological activities such as antitumor (inhibition of cancer cell growth, suppression of metastases, inhibition of beta transforming growth factor signaling) [19, 26, 27], anti-obesity on mice (inhibition of body fat accumulation, maintenance of normal body weight) [28], anti-leishmaniasis [25], and an inhibitory activity on the enzyme beta-secretase 1, a target in Alzheimer physiopathology [24].

In this study, we investigated whether 4-cholesten-3-one influences lipogenesis and cholesterol biosynthesis in two breast cancer cell lines, MCF-7 and MDA-MB-231. We also examined the effect of 4-cholesten-3-one on the integrity of membrane rafts to determine whether targeting of lipid metabolism can disrupt membrane rafts in cancer cells. Its ability to reduce cell viability and migration has also been studied.

## Materials and methods

### Materials

Two human breast cancer cell lines, MCF-7 and MDA-MB-231, and human monocyte THP-1 cells were obtained from the European Collection of Animal Cell Cultures (Salisbury, United Kingdom). 4-cholesten-3-one, Dulbecco’s Modified Eagle’s Medium (DMEM), Roswell Park Memorial Institute medium (RPMI 1640), Phorbol 12-myristate 13-acetate (PMA), 3-(4,5-Dimethylthiazol-2-yl)-2,5-Diphenyltetrazolium Bromide (MTT), fetal bovine serum, penicillin-streptomycin, trypsin, glutamine, dimethyl sulfoxide (DMSO), ethanol (EtOH), bovine serum albumin (BSA), primers for qPCR, triton X-100, OptiPrep™ Density Gradient Medium, SR9238, antibodies against beta-actin and EGFR, and 0.1% crystal violet solution were purchased from Sigma Aldrich (Saint-Quentin Fallavier, France). M-PER™ Mammalian Protein Extraction Reagent, Halt™ Protease and Phosphatase Inhibitor Single-Use Cocktail, and DAPI stock solution were obtained from Life Technologies (Saint-Aubin, France). TRIzol reagent for RNA isolation was from Invitrogen (Cergy-Pontoise, France). iScriptTM Reverse Transcription Supermix for RT-qPCR, and iQ™ SYBR Green Supermix were purchased from Bio-Rad (Marnes-la-Coquette, France). An antibody against flotillin-2 was obtained from Abcam (Paris, France). The IRDye whole IgG secondary antibodies, IRDye® 680RD Goat anti-Rabbit IgG (H + L) and IRDye® 800CW Donkey anti-Mouse IgG (H + L), were purchased from LI-COR Biosciences (Bad Homburg, Germany). 96 microwell plates (Nunc 160,376) were obtained from Nest Biotechnology Co., LTD (Rahway, USA). The Ibidi aqueous mounting medium was from Ibidi-cells in focus (Germany). Ambion® Silencer Selected Pre-designed, Validated Short-interfering RNA targeting LXRα (s19568) and LXRβ (s14684) (Ambion) were purchased from Thermo Fisher Scientific (Waltham, USA) as well as scrambled (non-targeting siRNA) negative control. Interferin was from Polyplus-transfection (Illkirch, France). The cell culture inserts were obtained from Falcon (Becton Dickinson, Oxnard, CA).

### Cell lines and culture

Breast cancer cells (MCF-7 and MDA-MB-231) and THP-1 cells were cultured in DMEM and RPMI 1640 respectively, both supplemented with 10% fetal bovine serum, 1% glutamine and 1% penicillin-streptomycin at 37 °C in a humidified atmosphere containing 5% CO_2_.

### Cells treatment

4-cholesten-3-one was dissolved in absolute ethanol to obtain a stock solution of 100 mM. For the treatment of cultured cells, the stock solution was diluted to the required concentration with a serum-free medium containing 0.1% BSA. The control cells were incubated with 0.2% EtOH.

### RNA extraction and real-time quantitative polymerase chain reaction (PCR)

Breast cancer cells were seeded at a density of 5 × 10^5^ cells/well in a 6-well plate and left to adhere overnight. THP-1 cells were plated at a density of 10^6^ cells/well in a 6-well plate and were allowed to differentiate into macrophage with 100 nM PMA for 24 h. Then, the culture medium was removed and the cells were treated for 24 h at 37 °C or transfected with siRNA targeting LXRα, LXRβ or a negative control siRNA, as described below. After 24 or 48 h of incubation, total RNA was extracted from cultured cells using TriZol Reagent according to the manufacturer’s recommendations. The concentration of the extracted RNA was determined by measuring the 260/280 absorbance with the NanoDrop ND-1000 spectrophotometer. 1 μg of total RNA was then reverse-transcribed into complementary DNA using iScript Reverse Transcription Supermix by following the manufacturer’s protocol. An initial priming step of 5 min at 25 °C was followed by a reserve transcription of 30 min at 42 °C and a reverse transcription inactivation step of 5 min at 85 °C. After cDNA synthesis, quantitative PCR was performed on a MyiQ2 Real-Time PCR Detection System (Bio-Rad) using an iQ™ SYBR Green Supermix. The cycling conditions were 95 °C for 30 s and 60 °C for 30 s for 45 cycles. The mRNA expression of FASN, ACC1, SCD1, HMGCR, ABCG1, ABCA1, LXR, APOE and 18S a housekeeping gene used as an internal control, was determined. The gene expression was normalized to the housekeeping gene using the 2^ΔΔCT^ method. The primer sequences used are shown in Table 1.

**Table 1:** Primer sequences used in this study

Gene symbol	Gene name	Sequence (5′-3′)
18S rRNA	18S ribosomal RNA	F- GATGCGGCGGCGTTATTCC R- CTCCTGGTGGTGCCCTTCC
ACC1	Acetyl-CoA Carboxylase 1	F- TCGCTTTGGGGGAAATAAAGTG R- ACCACCTACGGATAGACCGC
FASN	Fatty Acid Synthase	F- ACAGGGACAACCTGGAGTTCT R- CTGTGGTCCCACTTGATGAGT
SCD1	Stearoyl-Coenzyme A Desaturase 1	F- CAGAGGAGGTACTACAAACC R- ATAAGGACGATATCCGAAGAG
HMGCR	3-Hydroxy-3-Methyl-Glutaryl-Coenzyme A Reductase	F- TAACTCCTCCTTACTCGATAC R- AATAGATACACCACGCTCAT
ABCG1	ATP Binding Cassette Subfamily G Member 1	F- CAGGAAGATTAGACACTGTGG R- GAAAGGGGAATGGAGAGAAGA
ABCA1	ATP Binding Cassette Subfamily A Member 1	F- TCAGTGGGATGGATGGCAAAG R- TCCGACTCCGTCTGGCAATTA
LXRα	Liver X Receptor alpha	F- GCTCCCACCGCTGCTCTC R- TGCCCTTCTCAGTCTGTTCCAC
LXRβ	Liver X Receptor beta	F-ATCCACTATCGAGATCATGC R- GTCCTTCAAGAAGGTGATAC
APOE	Apolipoprotein E	F- CTGCGTTGCTGGTCACATTCC R- CGCTCTGCCACTCGGTCTG

### Lipid staining

MDA-MB-231 and MCF-7 cells were seeded at 2 × 10^4^ cells in 96 microwell plates and treated or not with 4-cholesten-3-one for 24 h. Cells were washed with PBS and fixed with 4% cold paraformaldehyde, then stained with Nile red (5 μg/mL) prepared in PBS from a stock solution at 0.5 mg/mL in DMSO. After incubation (30 min in the dark), the cells were washed with 1X PBS and then the cell nucleus was counter-stained (1:200) from a stock solution of DAPI 5 mg/mL diluted in PBS. Cells were washed and mounted using an Ibidi aqueous mounting medium. Fluorescence microscopy was performed using the IN Cell Analyzer 2200 (GE Healthcare, Vélizy-Villacoublay, France) with the Nikon focal lens (20X / 0.45). Nile red stains the lipid droplets and is observed using the red color channel (excitation at 542 nm, emission at 597 nm with an exposure time of 100 ms), whereas DAPI is observed in the blue color channel (excitation at 390, emission at 435 nm with an exposure time of 500 ms). Images were acquired with both detection channels and merged using ImageJ software.

### Protein extraction

MDA-MB-231 cells were grown in a 6-well plate at a density of 5 × 10^5^ cells/well and treated with 75 μM 4-cholesten-3-one for 24 and 48 h. Total proteins were extracted with lysis buffer composed of M-PER, protease and phosphatase inhibitors. The protein concentration was determined using the Bicinchoninic Acid Protein Assay.

### Western blot analysis

Proteins were separated by sodium dodecyl sulfate polyacrylamide gel electrophoresis (SDS-PAGE) and transferred to nitrocellulose membrane. The membrane was blocked with 5% skim milk in Tris buffered saline containing 0.1% Tween at room temperature for 2 h and then incubated at 4 °C overnight with primary antibodies against flotillin-2 (1/500), EGFR (1/1500) and beta-actin, a loading control (1/2000). Subsequently, the membrane was washed and incubated with IRDye whole IgG secondary antibodies (1/15000) at room temperature for 2 h. Finally, proteins bands were visualized using a B446 - LI-COR Odyssey® Infrared Imaging System.

### LXRα and LXRβ knockdown by small interfering RNA (siRNA)

Transient knockdown of LXRα and LXRβ by LXRα siRNA and LXRβ siRNA, respectively, was performed using Interferin according to the manufacturer’s instructions, whereas scrambled siRNAs were used in negative control cells. After 48 h of the transfection, the cells were used for further biological analysis.

### MTT assay

MCF-7 and MDA-MB-231 cells were plated at a density of 10^4^ cells/well in a 96-well plate and grown for 24 h. Thereafter, the cells were treated for 24 and 48 h at various concentrations of 4-cholesten-3-one (6.25, 12.5, 25, 50, 100 and 200 μM) and/or SR9238 (12.5 μM). For LXR knockdown analysis, the cells were treated with 4-cholesten-3-one (3.125, 6.25 and 12.5 μM) after siRNA transfection for 48 h. The effect of 4-cholesten-3-one on cell viability was examined using the MTT assay. After various times of treatment, 50 μL of MTT solution (2.5 mg/mL) was added to each well followed by 4 h of incubation at 37 °C. Then, the medium was removed and the formazan crystals were dissolved in 200 μL of DMSO. Finally, the absorbance was measured at 570 nm using a SpectraMax 190 microplate reader.

### Migration assays

#### Scratch wound healing assay

The scratch assay was performed to assess cell migration in vitro. MDA-MB-231 cells were seeded in a 6-well plate at a density of 10^6^ cells/well. When the cells reached confluency, a linear scratch wound was created in the middle of the confluent monolayer using a sterile 200 μL pipette tip. The culture medium was then removed and replaced with fresh medium supplemented with 4-cholesten-3-one or 0.2% EtOH as a control. The scratched area was captured at 0 and 48 h after scratching using an Olympus Inverted Phase Contrast Microscope with a 10X phase objective.

#### Transwell migration assay

A transwell migration assay was performed with an 8 μm pore size cell culture insert. The upper chamber of the transwell was inoculated with 1 × 10^5^ MDA-MB-231 cells and suspended in 200 μL of serum-free medium with BSA 0.1% DMEM with or without 4-cholesten-3-one (12.5 μM). The lower chamber was filled with 500 μL of DMEM containing 10% SVF. After 24 h of incubation, the membranes were fixed with 4% paraformaldehyde and stained with 0.1% crystal violet. The membranes were then photographed.

### Biochemical membrane raft isolation

The isolation of the membrane rafts was carried out as previously described by [29]. The breast cancer cells were plated at 2 × 10^6^ cells, incubated at 37 °C overnight and then treated with 4-cholesten-3-one at 50 μM for 48 h for MCF-7, and at 25 μM for 24 h for MDA-MB-231. Following incubation at 37 °C, the cells were washed with ice-cold phosphate-buffered saline (PBS) and lysed with 1 mL of lysis media containing 1% triton X-100 per condition. Next, the lysed cells (0.84 mL) were purified using an OptiPrep™ density gradient (35% (w/v), 1.16 mL) and placed in the bottom of a 10 mL ultracentrifuge tube. A discontinuous density gradient was then prepared by overlaying the three OptiPrep™ gradients (2 mL for each of the following gradients 30, 25 and 20%). The upper gradient layer consisted of 1 mL lysis buffer. Samples were centrifuged at 200,000×g for 4 h at 4 °C in a Beckman L8-70 M Ultracentrifuge, using TFT 65.13 rotor. Nine 1 mL fractions were collected from top to bottom of the gradient for each condition and then analyzed by western blot. The fractions corresponding to the membrane rafts are fractions 2, 3 and 4 isolated from MCF-7 cells and fractions 3, 4 and 5 isolated from MDA-MB-231 cells.

### Data analysis

Data represent mean values ± standard deviation of three independent experiments performed in triplicate. The Student’s t-test was used and the *p*-value < 0.05 was considered significantly different from the corresponding control.

## Results

### 4-cholesten-3-one reduces gene expression of key lipogenesis and cholesterol biosynthesis enzymes in breast cancer cells

**Fig. 2:**
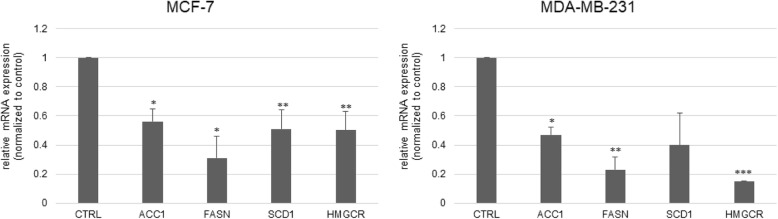
Expression levels of genes coding for enzymes of the lipogenesis and cholesterol synthesis pathway in breast cancer cells. MCF-7 and MDA-MB-231 cells were treated for 24 h with 4-cholesten-3-one at 50 μM. Total RNA was isolated and the mRNA expression of ACC1, FASN, SCD1 and HMGCR was examined by RT-qPCR. The mRNA expression was normalized to 18S rRNA and the results are expressed relative to the control set at 1. The data represent the mean ± the standard deviation. *p < 0.05, **p < 0.01, ***p < 0.001 as compared with control

Firstly, we determined whether treatment with 4-cholesten-3-one reduced lipogenesis and cholesterol biosynthesis in breast cancer cells. We therefore examined the mRNA expression of enzymes involved in these two pathways in MCF-7 and MDA-MB-231 cells treated or not treated with 50 μM 4-cholesten-3-one for 24 h. Compared with the control group set to 1, quantitative PCR results showed that 4-cholesten-3-one decreased the mRNA expression of the key lipogenic enzymes ACC1 (0.56 in MCF-7 and 0.47 in MDA-MB-231) and FASN (0.31 in MCF-7 and 0.23 in MDA-MB-231) (Fig. 2). The mRNA expression of SCD1 (0.51 in MCF-7 and 0.40 in MDA-MB-231), a key enzyme involved in the synthesis of monounsaturated fatty acids and HMGCR (0.50 in MCF-7 and 0.15 in MDA-MB-231), a rate-limiting enzyme for cholesterol synthesis, was also decreased in treated cells compared to untreated cells (Fig. 2).

**Fig. 3:**
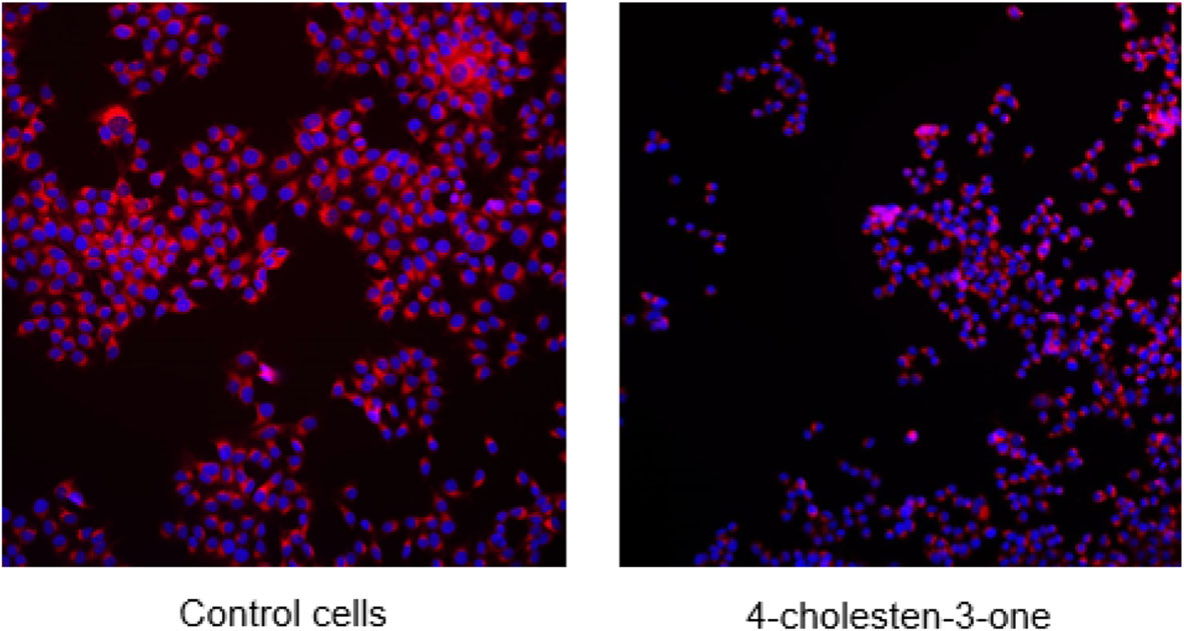
Effect of 4-cholesten-3-one on the accumulation of lipid droplets in MCF-7 cells. Cells were treated with 12.5 μM 4-cholesten-3-one for 24 h and stained with Nile red. Nile red staining corresponds to lipid droplets (red) and DAPI staining corresponds to the nucleus (blue)

We then studied lipid accumulation in MCF-7 cells following 4-cholesten-3-one treatment using Nile red staining which allows the visualization of lipid droplets. Figure 3 shows the cells treated or not with 4-cholesten-3-one, stained with Nile red solution and examined under a microscope. The stained lipid droplets decreased with 4-cholesten-3-one treatment compared to untreated cells.

### 4-cholesten-3-one increases the expression of ABCG1 and ABCA1 transporters in breast cancer cells

**Fig. 4:**
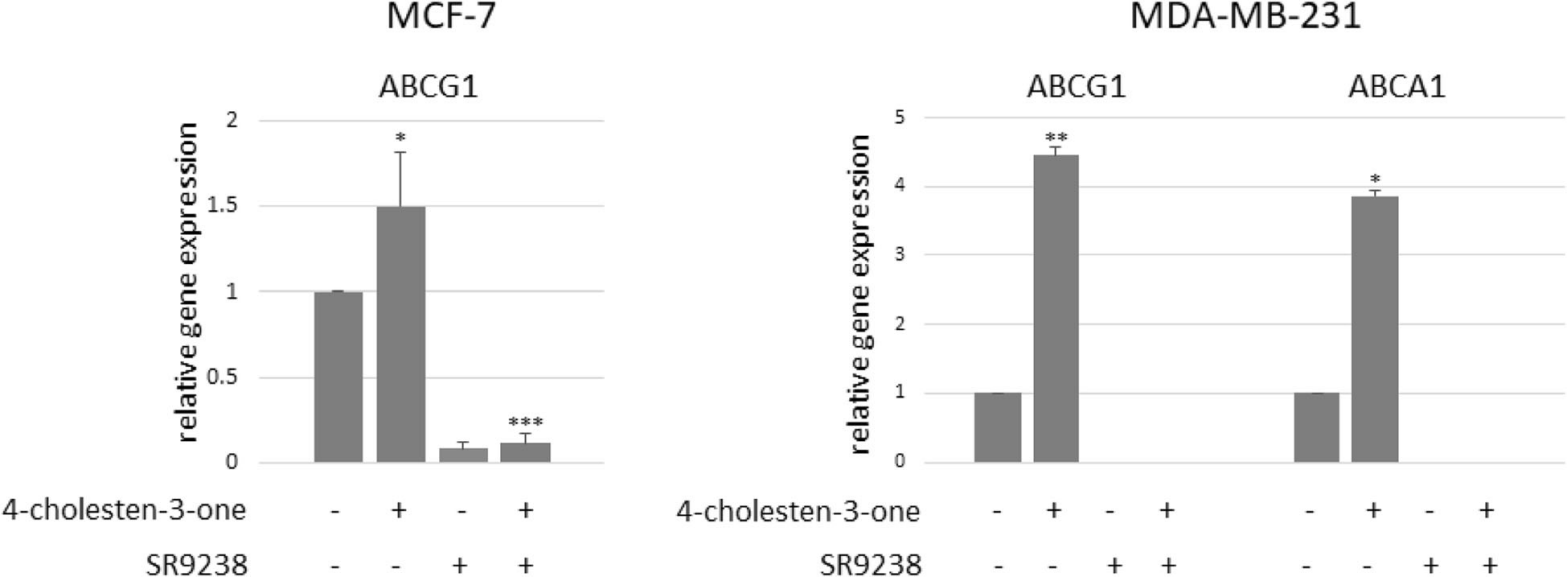
Effect of 4-cholesten-3-one and SR9238 on ABCG1 and ABCA1 mRNA expression level in MCF-7 and MDA-MB-231 cells determined by quantitative PCR. Breast cancer cells were treated for 24 h with 12.5 μM 4-cholesten-3-one alone, 12.5 μM SR9238 alone, or 12.5 μM 4-cholesten-3-one and 12.5 μM SR9238 together. 18S rRNA was used as a housekeeping gene and the results are expressed with respect to the control defined at 1. All data were presented as mean ± standard deviation. *p < 0.05, **p < 0.01, ***p < 0.001

RT-qPCR was then performed to examine the effect of 4-cholesten-3-one on the expression of ABC transporters, including ABCG1 and ABCA1. After 24 h of treatment, 12.5 μM 4-cholesten-3-one increased the mRNA expression of ABCG1 (1.50) in MCF-7 cells, and ABCG1 (4.46) and ABCA1 (3.86) in MDA-MB-231 cells compared to untreated cells (Fig. 4).

### Upregulation of ABCG1 and ABCA1 by 4-cholesten-3-one is suppressed by the LXR inverse agonist in breast cancer cells

To determine whether the increase in ABCG1 and ABCA1 expression induced by 4-cholesten-3-one was LXR-dependent, we decided to suppress LXR activity in breast cancer cells using an SR9238 inverse agonist and to subsequently study the mRNA expression of LXR target genes, ABC transporters. In MCF-7 cells, the mRNA expression of ABCG1 was significantly reduced to 0.08 when the cells were treated with SR9238 alone (12.5 μM) and to 0.11 after treatment with SR9238 (12.5 μM) and 4-cholesten-3-one (12.5 μM) together compared to untreated control cells after 24 h (Fig. 4). In MDA-MB-231 cells, SR9238 alone and the combination of SR9238 and 4-cholesten-3-one completely abolished the expression of ABCG1 and ABCA1 (Fig. 4).

### 4-cholesten-3-one increases mRNA expression of LXR and its target genes in THP-1 cells

**Fig. 5:**
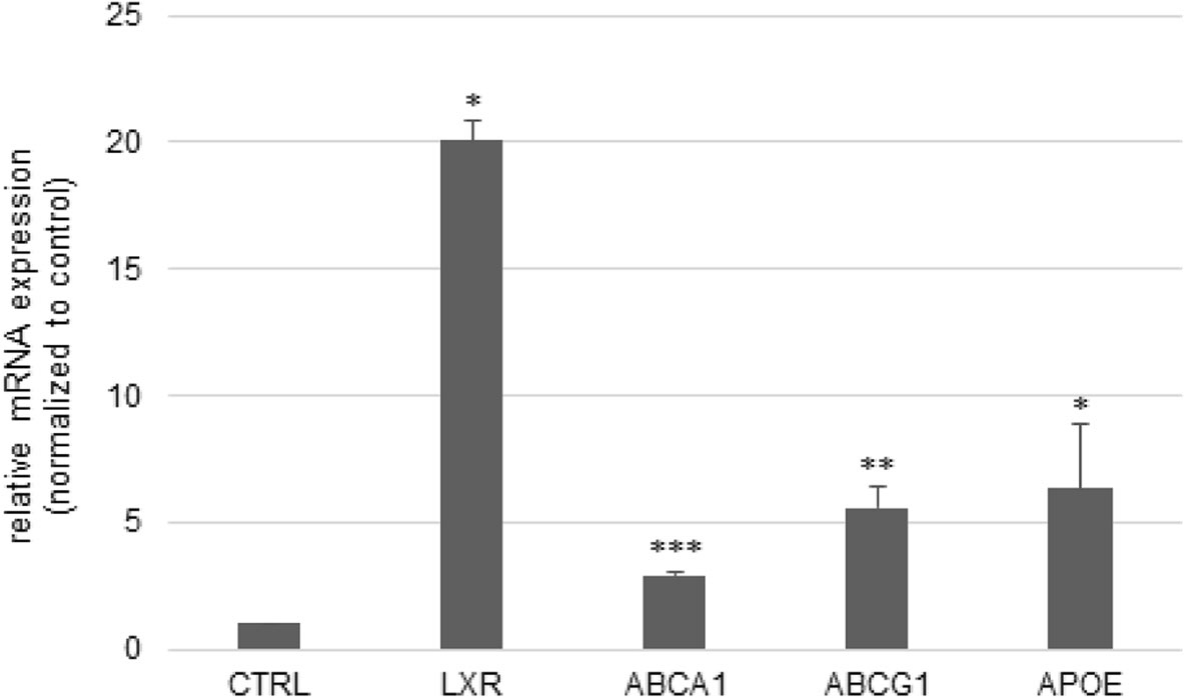
Effect of 4-cholesten-3-one on the mRNA expression of LXR and its target genes in THP-1 macrophages. THP-1 human monocyte cells were first differentiated into macrophage-like cells by exposure to phorbol 12-myristate-13-acetate and then treated with 12.5 μM 4-cholesten-3-one for 24 h. The mRNA expression was examined by Real-time RT-PCR. 18S rRNA was used as a housekeeping gene. Results are expressed as the means ± standard deviation. *p < 0.05, **p < 0.01, ***p < 0.001

The monocytic cell line THP-1 is frequently used as a macrophage cell model. Here, we tested the effect of 4-cholesten-3-one on the expression of LXR and its target genes. Treatment of macrophage-differentiated THP-1 cells with 12.5 μM 4-cholesten-3-one results in a remarkable increase in the mRNA expression of LXR (20.08) as well as its target genes ABCA1 (2.92), ABCG1 (5.59) and APOE (6.34) after 24 h compared to untreated cells (Fig. 5).

### 4-cholesten-3-one reduces the viability of breast cancer cells

**Fig. 6:**
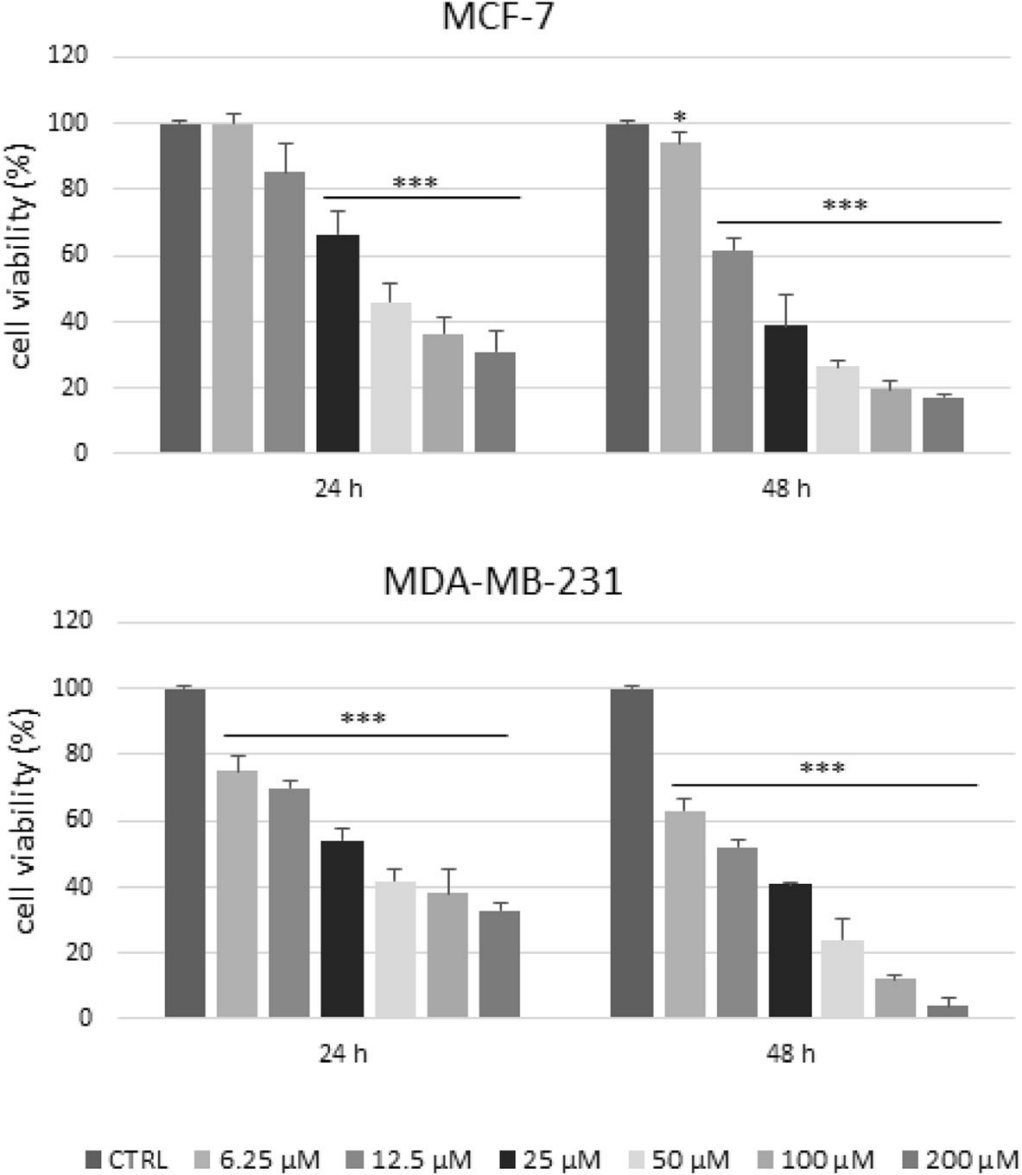
Cell viability of MCF-7 and MDA-MB-231 breast cancer cells after exposure to 4-cholesten-3-one. Cells were treated with the indicated concentrations of 4-cholesten-3-one for 24 and 48 h, and the cell viability was determined by MTT assay. The data are represented as mean ± standard deviation. Statistical analysis was performed using t-test, * denote *p* < 0.05, ** *p* < 0.01, *** *p* < 0.001

We then evaluated the effect of 4-cholesten-3-one on the viability of breast cancer cells. MCF-7 and MDA-MB-231 cells were treated with increasing concentrations (6.25–200 μM) for 24 and 48 h and cell viability was measured by MTT assay. As shown in Figs. 6, 4-cholesten-3-one reduced MCF-7 and MDA-MB-231 cancer cell viability in a dose-and time-dependent manner. The IC_50_ value was 17.8 and 14.1 μM after 48 h of treatment for MCF-7 and MDA-MB-231 cells respectively. The IC_50_ for the MCF-10A non-tumorigenic breast epithelial cell line was 60 μM after 48 h of treatment (data not displayed).

### Reduction of breast cancer cell viability by 4-cholesten-3-one is reduced by the LXR inverse agonist SR9238

**Fig. 7:**
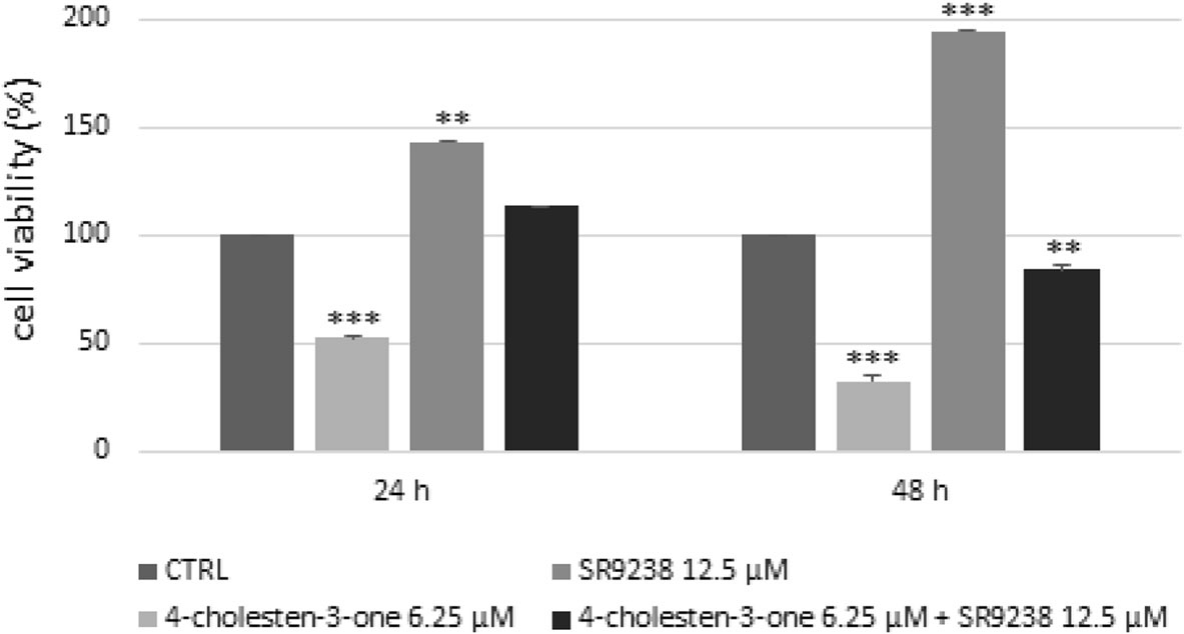
Effect of 4-cholesten-3-one and SR9238 on MCF-7 cells determined by MTT assay. Breast cancer cells were treated for 24 and 48 h with 6.25 μM 4-cholesten-3-one alone, 12.5 μM SR9238 alone, or 6.25 μM 4-cholesten-3-one and 12.5 μM SR9238 together. All data were presented as mean ± standard deviation. *p < 0.05, **p < 0.01, ***p < 0.001

SR9238 (12.5 μM) alone stimulated remarkably cell growth in MCF-7 cells after 24 and 48 h. As shown in Fig. 7, exposure of MCF-7 cells to 4-cholesten-3-one (6.25 μM) and SR9238 (12.5 μM) together resulted in decreased inhibition of cell viability compared to that obtained after treatment with 4-cholesten-3-one alone (6.25 μM). The results are unconvincing for MDA-MB-231 cells that were highly sensitive to 4-cholesten-3-one and less sensitive to SR9238 from the point of view of cell growth (data not shown).

### LXR knockdown rescues cell viability in cancer cells treated with 4-cholesten-3-one

**Fig. 8:**
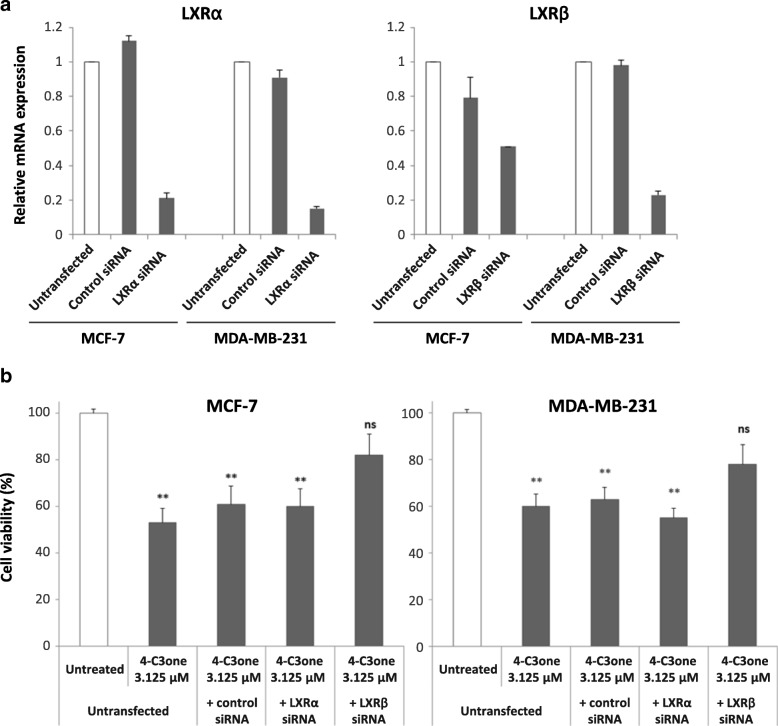
Effect of 4-cholesten-3-one on cell viability after LXRα and LXRβ knockdown. Cells were transfected or not with a siRNA specific for LXRα and LXRβ (20 nM) or a siRNA control. After 48 h of incubation, the cells were treated or not with 4-cholesten-3-one at the concentrations indicated. (**a**) LXRα and LXRβ relative mRNA expression determined by RT-qPCR after 48 h of transfection time. (**b**) Cell viability of MCF-7 and MDA-MB-231 cells after transfection and treatment with 4-cholesten-3-one. All data were presented as mean ± standard deviation. *p < 0.05, **p < 0.01, ***p < 0.001. ns means not significant

4-cholesten-3-one was incubated with cell lines MCF-7 and MDA-MB-231 transfected or not with a siRNA specific for LXRα or LXRβ. Validation of the LXRα or LXRβ knockdown was attested by an analysis of relative mRNA expression after 48 h transfection (Fig. 8a). As shown in Fig. 8b, the significant inhibition of cell viability induced by 4-cholesten-3-one at 3.125 μM was abolished in MCF-7 and MDA-MB-231 cells transfected with siRNA LXRβ. In contrast, 4-cholesten-3-one significantly decreased the cell viability of cells transfected with LXRα siRNA at the same levels as those used under control conditions (untransfected cells or cells transfected with non-targeted negative siRNA) in both cell lines.

### Interaction of 4-cholesten-3-one with the LXR binding domain

**Fig. 9:**
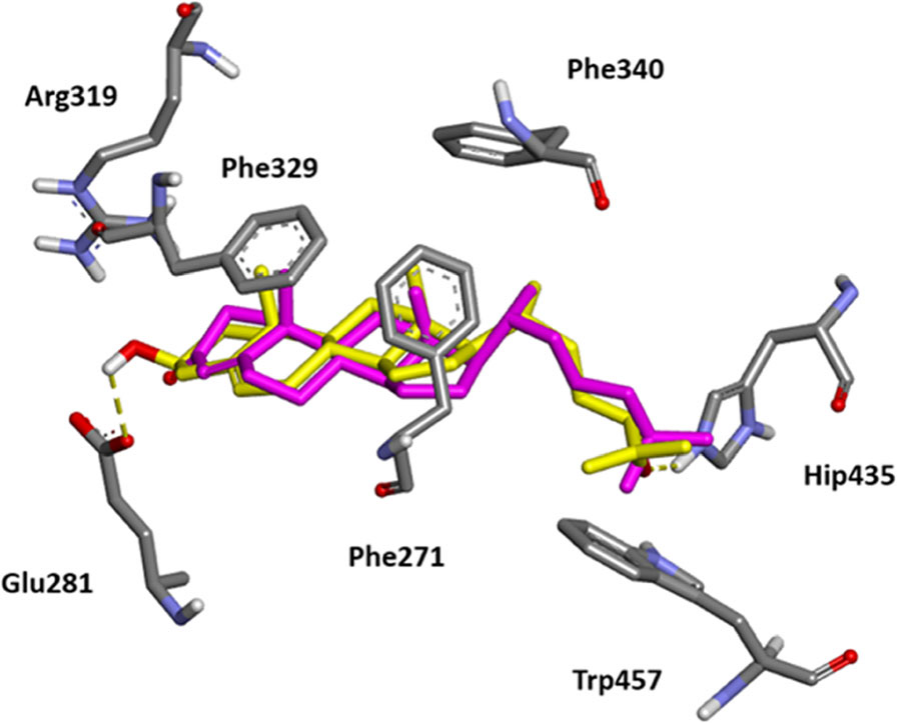
Comparison of the predicted binding mode for 4-cholesten-3-one with co-crystallized agonist 24(*S*),25-epoxycholesterol into the crystal structure of LXR*β* ligand binding domain (pdb code 1P8D). 24(*S*),25-epoxycholesterol is represented by a stick model (yellow, carbon atoms; red, oxygen atoms; white, hydrogen atoms). Predicted binding of 4-cholesten-3-one is represented by a stick model (magenta, carbon atoms; red, oxygen atoms). Hydrogen bonding interactions for 24(*S*),25-epoxycholesterol with key residues in the LBD (His435 (protonated form) and Glu281) are indicated as yellow dotted line

The binding pose found by the docking program GOLD (GOLD version 5.3.0; CCDC, Cambridge, UK) for 4-cholesten-3-one into the ligand binding domain (LBD) of LXR*β* in complex with 24(*S*),25-epoxycholesterol, a known agonist of LXR, is shown in Fig. 9. Both molecules adopt similar binding conformations with extensive lipophilic interactions (Phe271, Phe319, Phe340) into the LBD. The main difference is the lack of a key hydrogen-bond interaction with His435 which is observed with the epoxide oxygen of 24(*S*),25-epoxycholesterol and allowing an electrostatic interaction with Trp457 responsible for agonist conformation [30, 31].

### 4-cholesten-3-one reduces MDA-MB-231 breast cancer cell migration

**Fig. 10:**
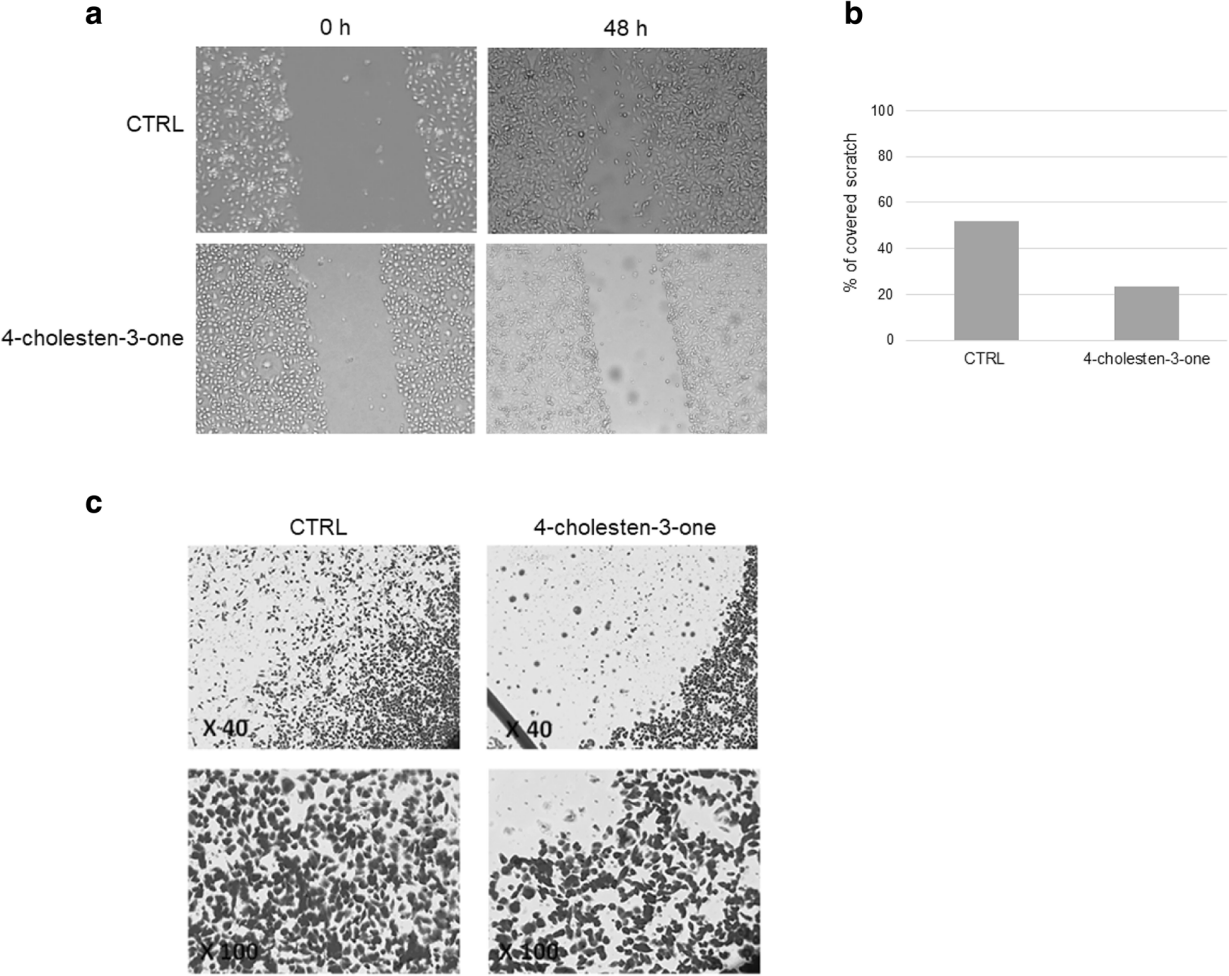
Effect of 4-cholesten-3-one treatment on MDA-MB-231 cell migration. *Wound healing assay*: cells were scratched and then incubated with 75 μM 4-cholesten-3-one for 48 h. Images were captured after the indicated times. (**a**) Representative images of cell migration. (**b**) The gap distance was quantitatively determined using MESURIM software after 48 h. The scratch width was measured as a percentage of the initial gap (the scratch width at 0 h was assumed to be 0% for both conditions). *Transwell migration assay*: cells were treated or not with 12.5 μM 4-cholesten-3-one and their migration capacity was determined by the Transwell assay after 24 h of incubation. (**c**) Representative images of the Transwell assay at × 40 and × 100 microscope magnification

We next examined the effect of 4-cholesten-3-one at 75 μM on MDA-MB-231 cell migration after 48 h of treatment using scratch wound healing assay and Transwell migration assay. Cells without treatment progressively reduced the width of the wound, a small sign of wound was observed after 48 h (Fig. 10a). Compared to cells in the control group, MDA-MB-231 cells exhibited reduced migration after treatment with 4-cholesten-3-one, the percentage of covered scratch was 23.4% after 48 h and the wound was not completely closed (Fig. 10a and b). These results were confirmed by the Transwell migration assay, as shown in Fig. 10c. Compared with untreated control cells, MDA-MB-231 cells exhibited reduced migration through transwell inserts after treatment with 12.5 μM 4-cholesten-3-one for 48 h.

### 4-cholesten-3-one disrupts membrane rafts of breast cancer cells

**Fig. 11:**
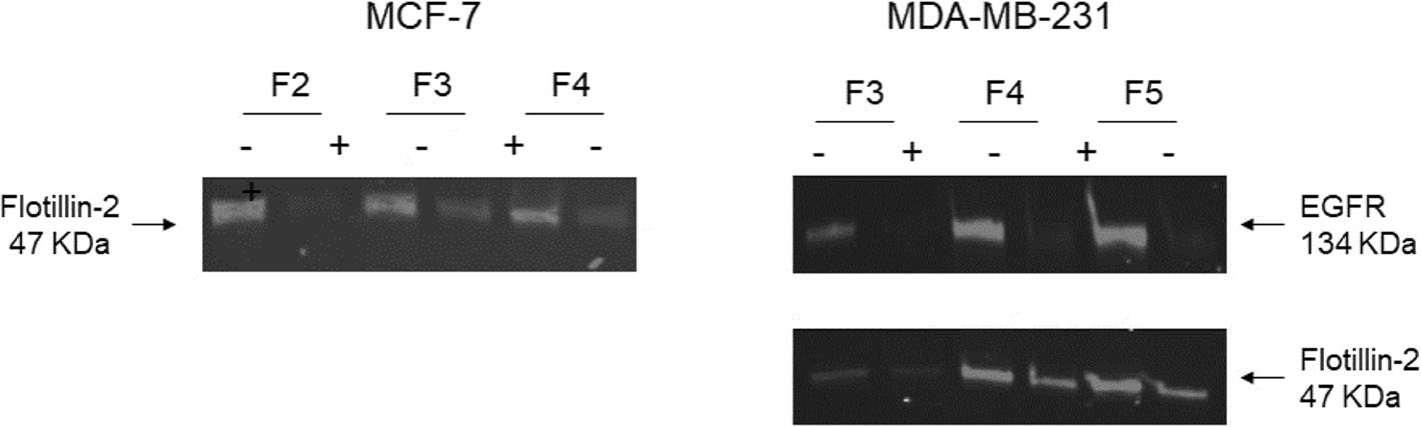
Effect of 4-cholesten-3-one on the expression of Flotillin-2 and EGFR in membrane raft fractions. Membrane rafts were isolated from MCF-7 and MDA-MB-231 cells as described in the Materials and Methods section. The membrane raft fractions were then collected, separated by SDS-PAGE and analyzed by western blot using anti-Flotillin-2 and anti-EGFR antibodies. A representative western blot showing Flotillin-2 and EGFR expression in cancer cells treated (+) or not treated (−) with 4-cholesten-3-one

To evaluate the effect of 4-cholesten-3-one on the membrane rafts of breast cancer cells, we first studied the protein expression of flotillin-2, a membrane raft marker. Western blot analysis showed that treatment with 4-cholesten-3-one resulted in decreased expression of flotillin-2 in fractions corresponding to membrane rafts (Fig. 11). We also observed a decrease in the level of EGFR protein in membrane raft fractions isolated from MDA-MB-231 cells treated with 4-cholesten-3-one as compared with those of untreated cells.

**Fig. 12:**
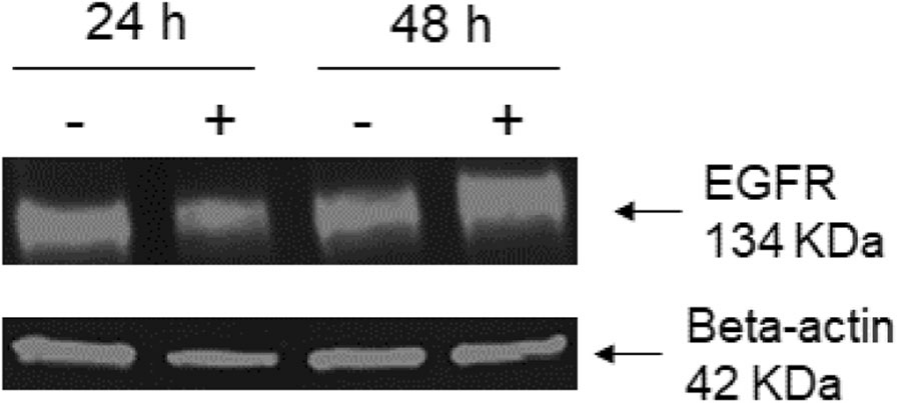
Expression of EGFR protein in whole lysates of MDA-MB-231 cells after treatment with 4-cholesten-3-one. Cells were treated or not for 24 and 48 h with 75 μM 4-cholesten-3-one. Western blot analysis was then performed to detect EGFR and beta-actin (loading control) in breast cancer cell lysates treated (+) or not treated (−)

Interestingly, we found that EGFR expression did not change in whole cell lysates after 24 and 48 h of MDA-MB-231 treatment with 75 μM 4-cholesten-3-one (Fig. 12).

## Discussion

4-cholesten-3-one results from the oxidation of cholesterol by cholesterol oxidase in the gastrointestinal tract [19]. It is found in bile, human gallstones and faeces [20, 21] and can also be identified in plant and animal tissues [22–25]. Recent work has shown that this intestinal cholesterol metabolite exhibits inhibitory activity of in vitro migration and metastasis in vivo in human lung adenocarcinoma cell lines [27], as well as inhibitory activity of transforming growth factor beta signaling in colorectal cancer cell line [19]. In this study, we sought to evaluate whether 4-cholesten-3-one exerts a cytotoxic effect on breast cancer cells while reducing lipogenesis and cholesterol biosynthesis and altering membrane rafts.

Increased lipogenesis is recognized as a hallmark of cancer. It is suggested that this process is necessary for cancer cell proliferation and survival [32, 33]. An upregulation of the enzymes involved in this pathway, such as ACC, FASN and SCD1, has been observed in various types of cancer (breast, prostate, …), and their inhibition in cancer cells leads to inhibition of cell proliferation and induction of apoptosis [32–36]. These lipogenic enzymes are therefore considered potential targets for cancer therapy. Since fatty acids are essential for cancer cell growth, we sought to evaluate the impact of 4-cholesten-3-one on the lipogenesis in breast cancer cell lines. We first examined the mRNA expression of the lipogenic enzymes in MCF-7 and MDA-MB-231 cells. Our results showed that 4-cholesten-3-one decreased the expression of ACC1, FASN and SCD1, indicating that 4-cholesten-3-one can reduce lipogenesis in breast cancer cells. According to the literature, Chajès et al. showed that silencing ACC and FASN genes in breast cancer cells resulted in decreased lipogenesis, leading to induction of apoptosis [37]. Likewise, several studies have indicated that ACC knockdown with siRNA induces apoptosis in prostate and breast cancer cells [32]. Moreover, FASN inhibitors (C75, cerulenin) and FASN siRNA treatments have been shown to induce apoptosis in breast cancer cells overexpressing FASN [38]. Inhibition of SCD1 expression in osteosarcoma, colon and breast cancer cell lines induces apoptotic cell death [39, 40]. Our data on lipogenesis were strengthened by our results with Nile red staining of breast cancer cells. Indeed, our work also provides evidence that 4-cholesten-3-one reduces the synthesis and storage of lipids in cancer cells by lipid droplet reduction after treatment. Lipid droplets are the sites where cells store excess lipids for their various biological functions [41]. High levels of lipid droplet in cancer cells have been shown to be associated with increased cancer aggression [42–44]. Finally, our results are consistent with the recently published article that shows that lithocholic acid, a cholesterol metabolite produced by intestinal bacteria, reduces lipogenesis and exhibits antiproliferative and pro-apoptotic effects in MCF-7 and MDA-MB-231 breast cancer cell lines [45].

High levels of intracellular cholesterol have been detected in cancers compared to normal tissues, particularly following the abnormal activation of the cholesterol synthesis pathway [46]. HMGCR, a rate-limiting enzyme essential for cholesterol synthesis, is upregulated in various types of cancer cells, including breast cancer cells, and plays an important role in cancer development [13]. Sánchez et al. [47] highlighted the antiproliferative effect of HMGCR inhibitors atorvastatin, fluvastatin and simvastatin on MCF-7 breast cancer cells; This effect was associated with a decrease in DNA synthesis and cell cycle arrest in the G1 and G2/M phases. A recent study of Ishikawa et al. [48] has shown that HMGCR inhibitor statin inhibits the proliferation and migration of cancer cells, as well as the formation of metastases. The effect of 4-cholesten-3-one on HMGCR expression was examined in MCF-7 and MDA-MB-231 cells and a decrease in mRNA expression was observed, suggesting that cholesterol synthesis was downregulated in our cell models treated with 4-cholesten-3-one. High cholesterol levels can also be explained by downregulation of ABC transporters, such as ABCG1 and ABCA1 [46]. This prompted us to study the expression of the genes involved in the cholesterol efflux, ABCG1 and ABCA1, after treatment with 4-cholesten-3-one. The breast cancer cells treated had a high expression level of these two ABC transporters after 24 h of treatment.

The liver X receptor (LXR), belonging to the nuclear receptor subfamily, plays a crucial role in the regulation of cholesterol homeostasis and lipid metabolism. Since ABCA1 and ABCG1 were reported to be under positive control of LXR, we decided to investigate whether the increase in transporter expression by 4-cholesten-3-one was dependent on LXR. The results showed that following treatment of breast cancer cells with 4-cholesten-3-one and SR9238 an LXR inverse agonist together, the mRNA expression of ABCG1 and ABCA1 was suppressed suggesting that the effect of 4-cholesten-3-one on ABC transporters expression is dependent on LXR in MCF-7 and MDA-MB-231 cells [49].

THP1 cells are widely used as a model for studying cholesterol efflux and the expression of LXR and its target genes. In order to confirm the results obtained on the expression of ABC transporters in breast cancer cells and whether 4-cholesten-3-one is an LXR agonist, macrophage-differentiated THP-1 cells were used. LXR expression was markedly increased in macrophage-differentiated THP-1 cells treated with 4-cholesten-3-one compared to control cells. The expression of the LXR target genes involved in the efflux of cholesterol ABCA1, ABCG1 and APOE, was also increased by 4-cholesten-3-one in THP-1 macrophages. These results indicate that 4-cholesten-3-one can act as an activator of LXR.

Recent studies have demonstrated the antiproliferative effect of LXR agonists in various cancer cell lines (prostate, breast, ovary, colon, leukemia) [11, 50]. We thus investigated biological activity of 4-cholesten-3-one in breast cancer cells. We showed that 4-cholesten-3-one exhibited a dose- and time-dependent cytotoxic effect on MCF-7 and MDA-MB-231 breast cancer cells with an IC_50_ value of 17.8 and 14.1 μM respectively after 48 h. This result is consistent with another study reporting the cytotoxic activity of 4-cholesten-3-one on several human breast, prostate and colon cancer cell lines [26]. Moreover, we found that 4-cholesten-3-one decreased MDA-MB-231 breast cancer cell migration which is in agreement with an earlier study in which it inhibited the migration of human lung adenocarcinoma cell lines [27]. To determine whether biological activity of 4-cholesten-3-one was LXR-agonist dependent, we used two approaches: co-treatment of cells with 4-cholesten-3-one and LXR inverse agonist, SR9238, and study of the activity of 4-cholesten-3-one after LXRα and LXRβ knockdown in our cell lines [49]. We thus first evaluated the effect of 4-cholesten-3-one, alone or in combination with SR9238, on the viability of breast cancer cells using the MTT assay. SR9238 treatment of MCF-7 clearly stimulated cancer cell growth. After treatment of these cells with 4-cholesten-3-one and SR9238 together, we noticed a decrease in the percentage inhibition of cell growth, demonstrating that the cytotoxic effect of 4-cholesten-3-one on MCF-7 cells may be dependent on LXR activation. Surprisingly, we did not observe an increase in MDA-MB-231 cell growth by SR9238 at the dose tested, suggesting that this breast cancer cell line is more resistant to LXR regulation than MCF-7 cell line. Moreover, we decided to knockdown the expression of LXR in our cancer cell lines using LXRα and LXRβ specific SiRNA. We hypothesized that if the 4-cholesten-3-one activity was due to its interaction with LXR, a decrease in LXR by siRNA knockdown would decrease its cytotoxic activity on our cell lines. Indeed, downregulation of LXRβ expression reduced the cytotoxic activity mediated by 4-cholesten-3-one. In contrast, we did not observe reduced activity of 4-cholesten-3-one after LXRα knockdown. Using docking studies, we have shown that 4-cholesten-3-one interacts with the ligand binding domain of LXRβ. Further experimentation needs to be performed in order to define the selectivity of 4-cholesten-3-one. However, these results were more likely to highlight the different roles of LXR isoforms in breast cancer cell lines in terms of survival and metabolic activities. LXR agonists such as T0901317 have been shown to decrease cell proliferation, increase expression of ABC transporters but also increase lipogenesis and levels of triacylglycerol in plasma and liver. For this reason, they have proven unsuitable for clinical trials [51]. In this study, we found that 4-cholesten-3-one acts as an LXR agonist, in particular by increasing the expression of ABC transporters, decreasing cancer cell viability. However, unlike the pan-LXR agonist, it significantly reduces lipogenesis.

Finally, we hypothesized that decreasing HMGCR and increasing ABCG1 and ABCA1 expressions involved in cholesterol biosynthesis and efflux, respectively, could disrupt membrane rafts. These latter are described as detergent-resistant microdomains of the cell membrane enriched in cholesterol and are involved in various cellular functions, including cell survival, proliferation and migration, which play an important role in cancer development and progression [13, 52]. Flotillin-2 is one of the membrane raft markers involved in cancer progression [16]. Moreover, a recent report of Carbonnelle et al. [53] showed that the LXR agonist, T0901317, which has been shown to have antiproliferative effect, alters the membrane rafts of MCF-7 cells as evidenced by the decreased expression of Flotillin-2. We therefore studied Flotillin-2 expression in MCF-7 and MDA-MB-231 breast cancer cells treated or not with 4-cholesten-3-one. Western blotting performed following membrane raft isolation revealed reduced expression of flotillin-2 in the membrane raft fractions of the two treated breast cancer cells, attesting that 4-cholesten-3-one could disrupt the integrity of membrane rafts.

Plasma membrane microdomains are also known to organize transmembrane receptors such as EGFR a member of the receptor tyrosine kinase family that is overexpressed in many cancer types such as breast and colon cancers [13, 14, 54]. Membrane raft fractions were found to contain more protein levels of EGFR than non-membrane raft fractions in cancer cells, such as cervical, colon and breast cancer cells [54]. In addition, membrane rafts disruption has been shown to alter EGFR signaling that affects various cellular processes in many cancers, including cell proliferation, apoptosis inhibition, cell migration, angiogenesis and metastasis [55, 56]. In this study, we found that treatment of MDA-MB-231 cells with 4-cholesten-3-one reduced EGFR expression in membrane microdomain fractions without altering EGFR expression in the whole cell lysate. It is known that EGFR is active especially when it is located in membrane rafts. Our results are therefore particularly interesting and highlight the ability of 4-cholesten-3-one to disrupt membrane rafts as well as the signaling molecules enriched in these microdomains.

## Conclusions

In conclusion, 4-cholesten-3-one is an original metabolite that acts as an LXR ligand without their adverse effect (enhanced lipogenesis). In this study, we showed that 4-cholesten-3-one exerts promising antiproliferative activity and inhibits migration of human MCF-7 and MDA-MB-231 breast cancer cells by (1) reducing lipogenesis and cholesterol biosynthesis (2) increasing the mRNA expression of the ABCG1 and ABCA1 transporters, and by (3) disrupting membrane rafts. With respect to its mechanism, it has been documented that 4-cholesten-3-one can replace membrane cholesterol [19, 27]. However, regarding our results, we can consider that (4) 4-cholesten-3-one also likely enters the target cells and interacts with LXR.

Taking everything into account, this study suggests that breast cancer cell viability and migration could be regulated by lipid metabolism pathway and membrane raft integrity under the control of 4-cholesten-3-one. We thus suggest a potential therapeutic role of 4-cholesten-3-one in breast cancer cells. Therefore, the nutritional intake of 4-cholesten-3-one or the induction of its production by intestinal bacteria could be a promising strategy for the prevention and/or treatment of breast cancer.

## Data Availability

All data generated or analyzed during this study are included in this manuscript.

## Abbreviations

ABCA1: ATP-binding cassette sub-family A member 1
ABCG1: ATP-binding cassette sub-family G member 1
ACC: Acetyl-CoA carboxylase
APOE: Apolipoprotein E
EGFR: Epidermal growth factor receptor
EtOH: Ethanol
FASN: Fatty acid synthase
HMGCR: 3-hydroxy-3-methyl-glutaryl-coenzyme A reductase
LXR: Liver X receptor
SCD1: Stearoyl-CoA desaturase 1
siRNA: small interfering RNA
